# Microcytosis Merits Evaluation: A Case Report of Hemoglobin S With Hereditary Persistence of Fetal Hemoglobin (HbS-HPFH Syndrome)

**DOI:** 10.7759/cureus.78827

**Published:** 2025-02-10

**Authors:** Claire E Daniel, Anthony Bejjani

**Affiliations:** 1 Addiction Medicine, Eisenhower Health, Rancho Mirage, USA; 2 Medicine-Pediatrics, David Geffen School of Medicine, University of California, Los Angeles, USA; 3 Hematology-Oncology, Veterans Affairs Greater Los Angeles Health System, Los Angeles, USA

**Keywords:** avascular necrosis, hbs-hpfh syndrome, hereditary persistence of fetal hemoglobin, microcytosis, osteonecrosis, osteonecrosis of the femoral head, sickle cell disease

## Abstract

Microcytosis is commonly encountered in primary care. Among patients with normal iron stores or compatible family history, hemoglobin electrophoresis, traditionally performed with gel electrophoresis and now through combination capillary electrophoresis/high-performance liquid chromatography, can diagnose thalassemic disorders or, rarely, a hemoglobinopathy. We present a case of an incidentally discovered microcytosis in a 34-year-old male patient where the workup led to a diagnosis of Hemoglobin S with Hereditary Persistence of Fetal Hemoglobin (HbS-HPFH syndrome). The diagnosis led providers to re-evaluate the patient’s chronic hip pain, upon which the patient was found to have bilateral osteonecrosis of the femurs.

Microcytosis is a lab abnormality that is often asymptomatic and, therefore, may be ignored or overlooked. When encountering microcytosis in primary care, it is important to identify the etiology using a brief, targeted approach. This may directly impact management and can prove helpful when considering future complaints of the patient.

## Introduction

Microcytosis is commonly encountered in primary care, often as an incidental finding. It most commonly occurs due to less hemoglobin in the cell [lower mean corpuscular hemoglobin (MCH)], which, in turn, is due to a lack of iron available for heme synthesis or defects in the globin chain synthesis. Microcytosis is quantified with a low mean cell volume (MCV) below the normal reference range, with reference ranges varying by laboratory. Iron deficiency and thalassemias, inherited disorders resulting in diminished alpha or beta globin chain synthesis, are the most common etiologies, with an increasing prevalence of thalassemia in the United States [1]. Other causes include thalassemia traits and disorders of iron utilization, including anemia of chronic disease (hepcidin-induced sequestration of iron), and rarely, sideroblastic anemia (inability to incorporate iron into the heme moiety). A brief, succinct evaluation is prudent since microcytosis may involve significant pathology. Most patients with microcytosis are asymptomatic, though laboratory values may suggest a diagnosis (e.g., new microcytosis is not compatible with a genetic cause). The measurement of serum iron, transferrin, and ferritin is generally the first step in evaluating microcytosis. In the case of iron deficiency, evaluating the source becomes a priority in the investigation. Among patients with normal iron studies or a compatible family history, gel hemoglobin electrophoresis has been used to rule in or rule out beta thalassemia subtypes by the detection of elevated levels of hemoglobin A2 (HbA2) [2]. Gel electrophoresis can also identify hemoglobinopathies and help identify the particular heritable cause for microcytosis or microcytic anemia. This is important, especially in young adults for whom genetic counseling is warranted [3]. Higher throughput and higher resolution methods such as capillary electrophoresis or high-pressure liquid chromatography (HPLC) are now used but retain the same diagnostic algorithm and interpretations. A specific diagnosis that one may achieve through hemoglobin fractionation, as illustrated in the present case, may also contextualize the patient’s future medical complaints. We present a case of incidentally discovered microcytosis where the workup led to a diagnosis of Hemoglobin S with Hereditary Persistence of Fetal Hemoglobin (HbS-HPFH syndrome) and osteonecrosis of the femurs.

The learning objectives from this case are to recognize microcytosis as a clinically significant abnormality and to utilize an algorithm for diagnosing unexplained microcytosis.

## Case presentation

A 34-year-old man was evaluated at the hematology clinic of the West Los Angeles Veterans Affairs Medical Center because of an abnormal hemoglobin electrophoresis. Six months prior, a complete blood count (CBC) was performed for the evaluation of acute diarrhea. The acute diarrhea resolved itself, and the CBC was normal aside from a low MCV of 77.9 (reference range: 80.0-100.0). All the relevant laboratory results are shown in Table 1.

**Table 1:** The patient's laboratory results *At the West Los Angeles Veterans Affairs Medical Center; Hemoglobin fractionation was performed by gel electrophoresis at Quest Diagnostics.

Variable	Reference range in adults*	6 months prior (primary care)	4 months prior (primary care)	On evaluation (hematology clinic)
White-cell count (per μl)	4.5-11.0	7.73	-	8.96
Hemoglobin (g/dl)	Male: 13.3-17.7, Female: 11.7-15.7	15.3	-	14.4
Hematocrit (%)	39-52	45.4	-	42
Platelet count (per μl)	150-440	175	-	198
Mean corpuscular volume (fl)	80-99	77.9	-	75.7
Mean corpuscular hemoglobin (pg)	27-34	26.2	-	25.9
Mean corpuscular hemoglobin concentration (g/dl)	32-36	33.7	-	34.3
Red-cell distribution width (%)	12 to 15	15.7	-	15
Reticulocyte count (%)	0.5-1.5	-	-	2.35
Lactate dehydrogenase (U/liter)	87.0-271	145	-	-
Ferritin (μg/liter)	Male: 22-322, Female: 10-291	128.1	-	-
Transferrin (mg/dL)	200-370	285	-	-
Calculated total iron binding capacity (μg/dL)	240-400	382.4	-	-
Iron (μg/dL)	50-212	103	-	-
Hemoglobin A (%)	> 96.0	-	0	-
Hemoglobin F (%)	< 2.0	-	35.3	-
Hemoglobin A2, QUANT (%)	0-4.5	-	2.2	-
Hemoglobin S (%)	No reference range	-	62.5	-
Sodium (mmol/liter)	136-146	140	-	135
Potassium (mmol/liter)	3.5-5.3	4.6	-	4.1
Chloride (mmol/liter)	95-110	102	-	103
Carbon dioxide (mmol/liter)	21-31	29.7	-	23.1
Urea nitrogen (mg/dl)	May-25	13	-	14
Creatinine (mg/dl)	Male: 0.66-1.28, Fem 0.52-1.04	1.06	-	1.06
Glucose (mg/dl)	70-110	86	-	95
Albumin (g/dl)	3.2-4.8	4.1	-	-
Total protein (g/dl)	5.9-8.3	7.1	-	-
Alanine aminotransferase (U/liter)	7.0-45.0	48	-	-
Aspartate aminotransferase (U/liter)	13-35	21	-	-
Alkaline phosphatase (U/liter)	33-94	64	-	-
Total bilirubin (mg/dl)	0.2-1.0	0.8	-	-

Further testing was performed to evaluate the etiology of microcytosis. Serum ferritin was normal, as were iron, total iron-binding capacity (TIBC), and transferrin saturation. A review of prior CBCs showed consistent microcytosis with an MCV range of 75.4-77.9 femtoliters during the preceding seven years.

**Figure 1:**
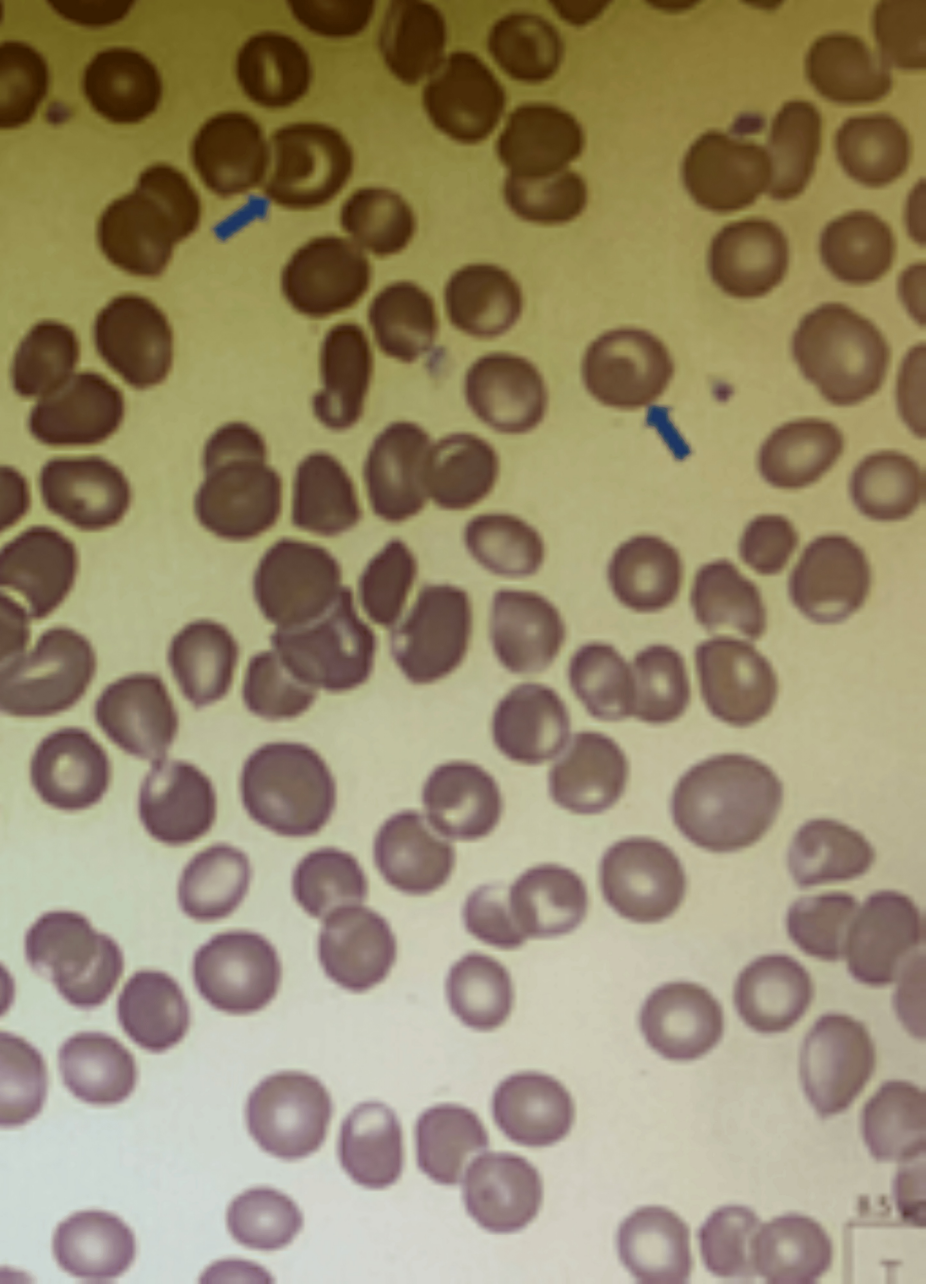
The peripheral blood smear shows numerous target cells (blue arrows) but is otherwise unremarkable Target cells are also known as codocytes, a term derived from the Greek word for hat. When these cells are spread onto a glass slide, a central bump of hemoglobin appears as a target. This is considered as a manifestation of excess cellular membrane relative to the hemoglobin inside.

Two months later, and four months before the hematology evaluation, hemoglobin electrophoresis was performed to evaluate for suspected thalassemia. It returned with hemoglobin A (HbA) of 0%, hemoglobin S (HbS) of 62.5%, hemoglobin F (HbF) of 35.3%, and HbA2 of 2.2%, clinically consistent with homozygous sickle cell disease (HbSS) with hereditary persistence of fetal hemoglobin (HPFH). The peripheral blood smear showed numerous target cells and was otherwise unremarkable (Figure 1).

Lactate dehydrogenase and bilirubin levels were both normal (Table 1). The patient was then referred to the hematology clinic of this hospital.

The patient denied frequent infections, though he had been to the emergency room for viral gastroenteritis three times in the preceding year. He reported a history of asthma as a child but had no respiratory complaints as an adult. He had a 10-year history of intermittent low back pain with radiation to the legs and hip that had required opioid treatment in the past and multiple ED visits for recurrent flares, with no imaging within this hospital in the preceding two years. He also had prior bilateral hip pain with mild arthritis on X-rays performed two years prior to presentation. He was followed by the chronic pain clinic and had received injections and radio-ablation for a diagnosis of lumbar discogenic pain. 

His medical history otherwise included vitamin D deficiency and post-traumatic stress disorder related to his military service. He took no other medications. There was no pertinent surgical history.

The patient identified himself as an African-American man. He operated a forklift overnight at a large warehouse. He was an army combat veteran in reserves and had been deployed previously to Iraq and Kuwait. He drank two glasses of wine a month. He had a history of tobacco use during deployment and smoked cigars only on significant occasions. There was no other drug use.

His family history included sickle cell trait in his mother, diagnosed many years ago as per the patient's history. The patient's two maternal cousins had sickle cell anemia. His mother was Guatemalan and his father was reportedly African-American, but he did not know his father’s medical history, and multiple requests made by him for his father to have additional testing did not result in further testing. The patient did not have any siblings.

On examination, vital signs were normal, as were an examination of the head, thorax, and abdomen. There was positive Trendelenburg sign bilaterally and positive Faber test on the right. The range of motion in both hips was limited by pain.

To further elucidate the mechanism of microcytosis, the seven most common alpha globin chain mutations and thalassemia-causing beta globin chain mutations were tested and were negative.

**Figure 2:**
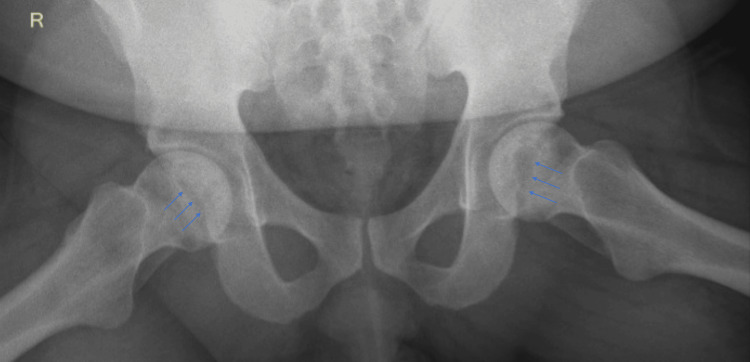
X-ray of the pelvis with serpiginous sclerosis with central lucencies at the femoral heads (blue arrows) suggesting osteonecrosis

To assess for potential complications of sickle cell disease, the patient underwent abdominal ultrasound which showed a normal spleen and no gallstones. He was referred to ophthalmology for retinopathy screening, which was normal. Upon learning of his hemoglobin electrophoresis results, the patient revealed multiple other areas of joint pain that occurred during winter months for years. He expressed concern that they were related to his sickle cell disease. None of these episodes required emergency room care. The electrophoresis results also merited re-evaluation of his hip pain and raised concern for possible osteonecrosis (OST), a well known complication of sickle cell disease [4,5]. Plain films of the hips and femurs showed serpiginous sclerosis with central lucencies at the bilateral femoral heads (Figure 2) suggesting OST.

**Figure 3:**
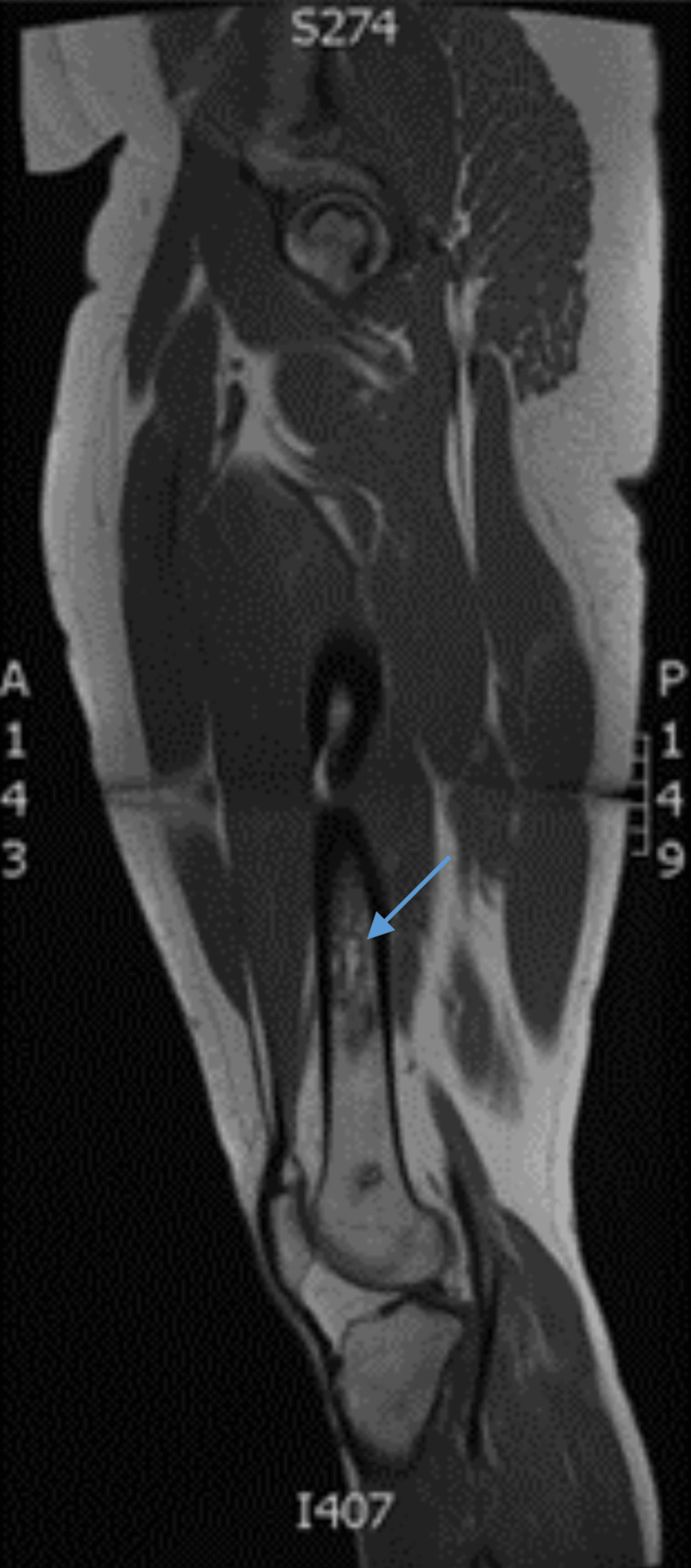
A saggital 1.5 Tesla MRI, T1 turbo-spin echo demonstrated osteonecrosis of the left femoral head occupying approximately 40% of the weight-bearing surface and an elongated bone infarct within the left mid-femoral diaphysis (blue arrow). Additional smaller bone infarcts are visible at the distal femur.

Subsequent MRI confirmed bilateral femoral head OST without joint collapse and also showed elongated bone infarcts in both femurs (Figure 3).

Given the patient's pain episodes during the cold months, despite lack of evidence for hydroxyurea stemming osteonecrosis progression, the patient started on hydroxyurea 15 mg/kg/day towards the end of fall after consultation with other practicing hematologists and with shared patient decision making. He noted subjective improvement in his hip and knee pain, and reported no pain episodes during that subsequent winter. He was referred to orthopedic surgery for evaluation and surveillance of his osteonecrosis, given the high likelihood of the need for future hip replacement. He was sent to ophthalmology for annual retinopathy screening. Three months after initiating hydroxyurea, he moved away to be closer to his family. Further titrations or adjustments of hydroxyurea were to be made by his subsequent hematologist. It was recommended that his father be screened with electrophoresis for sickle cell trait and beta thalassemia in order to estimate the probability of his carrying two HbS variants, rather than one. Family planning was discussed, with the recommendation to screen his partner for sickle cell trait.

## Discussion

Microcytosis results from insufficient heme or globin synthesis during red cell production. Iron is a component of heme, and its deficiency will produce microcytosis. Thalassemias are disorders of reduced or absent globin chain synthesis, whereas hemoglobinopathies affect the structure of the globin chains. Microcytosis is a feature of thalassemia and some hemoglobinopathies, such as hemoglobin C (HbC) disease. Following the prompt ruling out of iron deficiency, hemoglobin fractionation may be warranted to diagnose a cause for persistent microcytosis. 

This patient presented with microcytosis without iron deficiency and was found to have predominantly HbS (α_2_β^S^_2_), high levels of HbF (α_2_γ_2_), and absent HbA (α_2_β_2_). This pattern is consistent with either HbSS or HbS-β^0^-thalassemia, as both conditions result in an absence of HbA. The normal value of HbA2 raises the possibility of delta-beta thalassemia (HbS/δβ^0^-thalassemia), where the HbA2 is low or normal owing to the deletion of the delta-globin gene. Traditionally, the diagnosis of HbS/β^0^-thalassemia requires HbA2 >3.5%, which was not the case in this patient [6].

It is important to remember that microcytosis is not typically a feature of HbSS disease [7]. This patient’s microcytosis ruled in concomitant thalassemia, that is, insufficient hemoglobin produced per cell. A mutation in one alpha globin allele (1/4, silent carrier) or two alpha globin alleles (2/4, trait) does not lead to the production of abnormal hemoglobin chains, and the electrophoresis results will be normal. Testing for alpha globin chain mutations remains the definitive test for those in whom a clinical diagnosis of alpha thalassemia trait cannot be made. For this patient, the seven most common alpha globin chain mutations were tested and were negative.

Common thalassemia-causing beta globin mutations were also tested and were negative. Specifically, an analysis was performed for the promoter region, all three exons, a portion of intron 2, and the splice sites of the hemoglobin subunit beta (HBB) gene. These negative tests could not rule out a heterozygous large deletion involving all or part of the HBB gene, such as delta-beta thalassemia or deletional HPFH. This suggested three possibilities: HbSS with an uncommon (and therefore untested) alpha globin chain mutation; HbS/δβ-thalassemia; and HbS with HPFH with an incomplete increase in gamma globin chain production (HbS-HPFH syndrome). In the present case, the absence of anemia and the patient’s relatively benign clinical course made HbS-HPFH syndrome the most likely diagnosis [8].

HPFH can be divided into two broad groups: deletional and non-deletional. Non-deletional HPFH primarily involves mutations of the gamma globin promoter region, such that gamma globin synthesis is relatively under-suppressed into adulthood [9]. Mutations in this region that affect the binding sites for the HbF repressors ZBTB7A and BCL11A are examples [10]. By comparison, deletional HPFH refers to variable-length deletions of the beta-globin locus on chromosome 11 that lead to absent beta globin synthesis [6]. Deletions in the beta gene locus are associated with variable compensatory increases in the expression of the gamma globin gene, which also sits on chromosome 11. Deletional HPFH is associated with uniform microcytosis, suggesting that the compensatory increase in the gamma globin chain production creates insufficient hemoglobin to allow for normal red cell size and thus creates very mild thalassemia [10].

In the present case, the patient’s uniform microcytosis is most consistent with a deletional HPFH genotype. We did not have access to other laboratory testing that could confirm the precise mechanism of HPFH, although one can test specifically for HPFH-causing deletions and sequence the promoters of the gamma-globin genes to look for uncommon HPFH point mutations in transcription factor binding domains. As a whole, the level of HbF observed among patients with deletional HPFH is higher than among those with non-deletional HPFH and compatible with the 35.3% observed in this patient [9,10]. In this patient with HbS, the HbF being elevated to 35.3% was still insufficient for a completely benign clinical course and to allow a normal MCV. However, the presence and persistence of high percentages of HbF help prevent significant amounts of hemolysis. The distribution of HbF across cells is one factor that can affect the clinical phenotype of patients with HbS-HPFH, and its discussion is beyond the scope of this report but is reviewed elsewhere for those interested [10].

The clinical phenotype of patients with HbS-HPFH, though often described as “benign”, is poorly characterized due to the rarity of the condition and a paucity of clinical information from case series [10,11]. There are documented cases of osteonecrosis, splenic infarction, retinopathy, and hemiparesis among patients with HbS-HPFH [12,13]. Most patients in published case series are young, which may lead to underrepresentation of age-related complications of sickle cell disease.

The present case highlights the breadth of genetic conditions that cause microcytosis and the importance of diagnosis. This patient was appropriately evaluated with capillary electrophoresis after the possibility of iron deficiency had been eliminated. As a result, he was spared a potentially inappropriate iron prescription. Occasionally, empiric iron therapy is started for microcytosis without further evaluation. The documentation of thalassemia in the problem list can avoid this low-value practice. Additionally, confirming thalassemia, even if it is thalassemia minor or trait, would aid in family planning. Hematologists may help delineate which patients would benefit most from globin chain mutational testing and subsequently which ones should be prioritized for genetic counseling. Referral to a hematologist is also indicated for assistance with symptomatic hemoglobinopathies or thalassemia.

The patient's diagnosis also led to relevant screenings for sickle cell retinopathy and hyposplenism, and led providers to recontextualize his low back and hip pain. His pain, previously attributed to lumbar disc degeneration, now mandated inclusion of osteonecrosis, a characteristic complication of sickle cell disease, in the differential diagnosis.

## Conclusions

Microcytosis is a laboratory abnormality that is often asymptomatic and therefore may be ignored or overlooked. When encountering microcytosis in primary care, it is important to identify the etiology using a systematic approach, as it may directly impact management. An evaluation for iron deficiency, typically using serum ferritin, is the essential first step. Among patients with normal iron stores or a compatible family history, hemoglobin electrophoresis is recommended to evaluate for thalassemia and other hemoglobinopathies. Diligent documentation of family history may provide a further impetus to promptly assess chronic microcytosis. The diagnosis of an underlying genetic cause informs patient care and counseling on family planning and prevents unnecessary iron prescriptions. It can also expand the differential diagnosis for subsequent symptomatology, as it did for this patient’s hip pain. Genetic disorders that cause microcytosis may be clinically significant, even in the absence of anemia.
